# Fine needle aspiration of lung lesion: Lateral and transpectoral approach

**DOI:** 10.2349/biij.8.1.e2

**Published:** 2012-01-01

**Authors:** K Smith

**Affiliations:** Radiology SA, Calvary Central Districts Hospital, Elizabeth Vale, South Australia, Australia.

**Keywords:** Fine needle aspiration (FNA), pneumothorax, lung lesion, biopsy, CT

## Abstract

A 69 year-old man presented with an incidental finding on radiograph of a lesion in the left upper lobe. CT indicated it was likely to be a neoplasm and CT-guided FNA was requested. The lesion was located medial to the scapula so a creative approach was utilised to gain access to the lesion. This study discusses the approach used and why it reduced patient risk compared to a more conventional procedure. The sample was positive for neoplasm and there were no complications arising from the procedure.

## INTRODUCTION

Lung biopsy has been performed since as early as 1883 [1], although with poor results in many of the early cases. Avoiding the procedure in patients who have significant contraindications has led to increased safety. These contraindications [2] include:

- Poor lung function/respiratory failure
- Pulmonary hypertension
- Abnormalities of coagulation
- Poor patient cooperation

Pulmonary lesions are often difficult to localise with conventional radiographic or fluoroscopic techniques. CT-guided fine needle aspiration (FNA) has become the method of choice for a number of reasons. The soft tissue differentiation of CT allows the radiologist to plan an approach which avoids important underlying structures like blood vessels and lymph nodes. Accurate measuring software creates an opportunity to set needle entry points and angles, and to gauge the depth required for an effective sample to be acquired. The development of multi-slice CT has improved the accuracy and speed of interventional imaging by allowing multiple slice acquisition without the need for patient table movement. This allows the interventionalist to see the length of their needle when it is not parallel to the scan plane and therefore adjust its position more instinctively.

Less than half of the solitary pulmonary nodules detected on CT are malignant; of these, 15% are small cell carcinomas (SSC) and 85% non-small cell carcinomas (NSCC). The NSCC group is further divided into three groups; adenocarcinoma, squamous cell carcinoma, and large cell carcinoma, with large cell carcinoma making up 10–15% of all lung cancers. All three share similarities in behaviour and treatment [3].

## HISTORY

**Figure 1:**
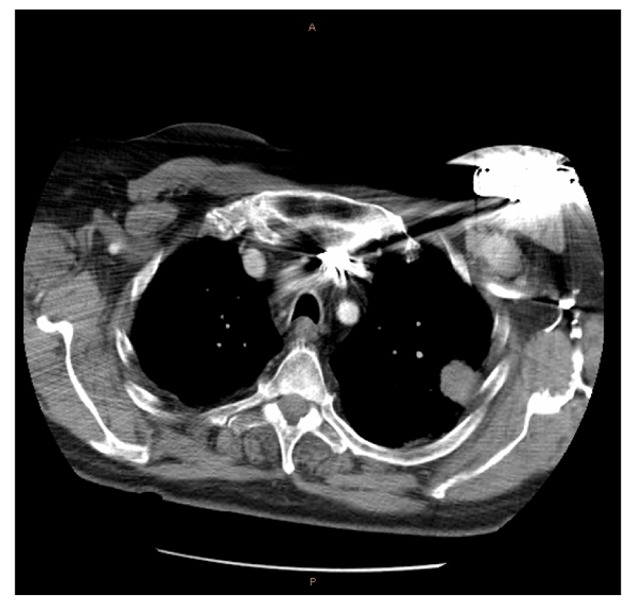
This image indicates the location of the lesion in relation to the anatomical structures. The difficulty caused by the scapula is apparent.

A 69 year-old man with mild to moderate Chronic Obstructive Pulmonary Disease (COPD) and a previous diagnosis of Left Ventricular Failure (LVF) with an implanted defibrillator presented to the Medical Imaging Department in October 2010. Previous imaging in another department included a routine chest radiograph which revealed an opacity of 32 mm × 22 mm in the left upper lobe (Figure 1).

Further CT imaging strongly suggested this was neoplastic and required CT-guided FNA. The CT report described the lesion as sub-pleural, abutting the pleura and technically difficult for FNA. The lesion's postero-lateral location placed it directly medial to the scapula in the routine supine CT position. Although he had COPD, the patient did not have any of the other contraindications for the procedure discussed in the introduction. In view of the sub-pleural location of the lesion, it was not accessible bronchoscopically and the radiologist decided to perform the procedure.

## MATERIALS AND METHOD

After consultation with the radiologist, the patient was placed in a right decubitus position, feet first in the gantry of a 64-slice Siemens Definition CT scanner manufactured in Erlanger, Germany and installed in April 2010. Foam positioning aids were used to support this position. The patient's left hand was placed so the palm was on his left superior buttock. He was asked to pull his elbow backwards and medially so as to move the shoulder posteriorly. This position moved the anterior surface of the shoulder back allowing a transpectoral path to the posterior, apical biopsy site. The use of such an unconventional position provided access to the lesion while avoiding the axilla which contains the axillary vessels, and the brachial plexus of nerves, with their branches, some branches of the intercostal nerves, and a large number of lymph glands, together with a quantity of fat and loose areolar tissue. The axillary artery and vein, with the brachial plexus of nerves, extend obliquely along its lateral boundary [4]. The use of this position and its increased patient safety is the focus of this case study. A literature review did not reveal journal articles or textbooks documenting this method of approach. A section of 8FG feeding tube was taped to the patient running from the clavicle to the nipple level to provide a reference marker for marking an entry point.

**Figure 2:**
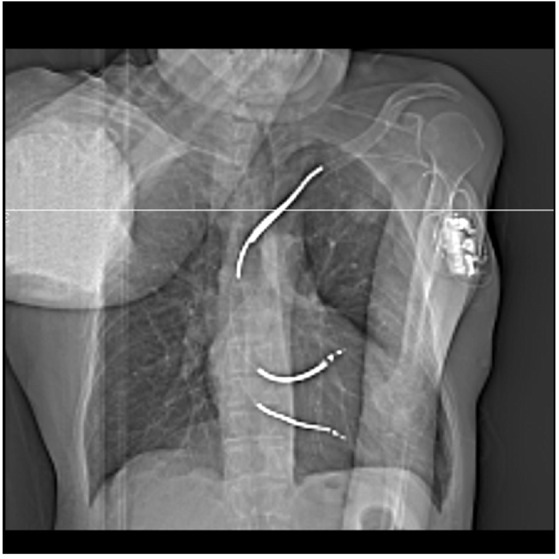
The lesion is seen on the CT topogram with a defibrillator in situ anteriorly and the scapula limiting access posteriorly.

**Figure 3:**
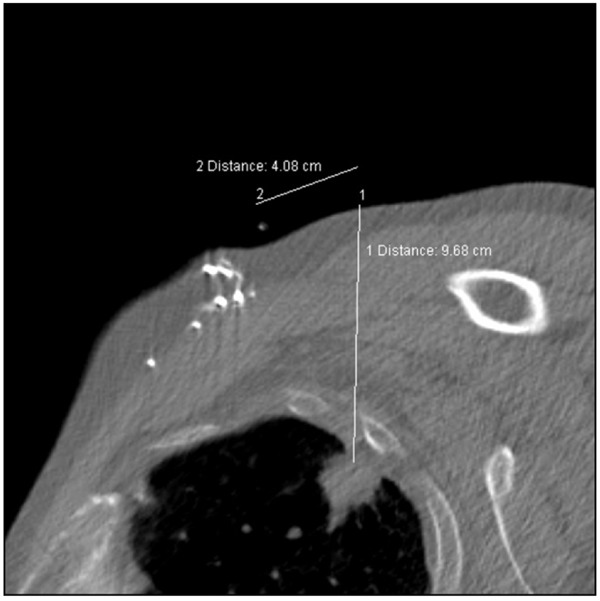
With the patient in the right lateral decubitus position, the scapula is posterior to the lesion and adequate access is available via the pectoral muscle and axilla. Measurements from the skin marker indicate position of entry and the needle depth required.

A topogram was performed with the CT tube in a lateral gantry position to provide an AP chest type image (Figure 2) and a series of 3 mm axial scans was performed to encompass the lesion. This scan series indicated that in this position, the lesion was accessible from a point anterior to the shoulder. Measurements were taken against the skin reference and an entry point was marked with a permanent marker (Figure 3).

**Figure 4:**
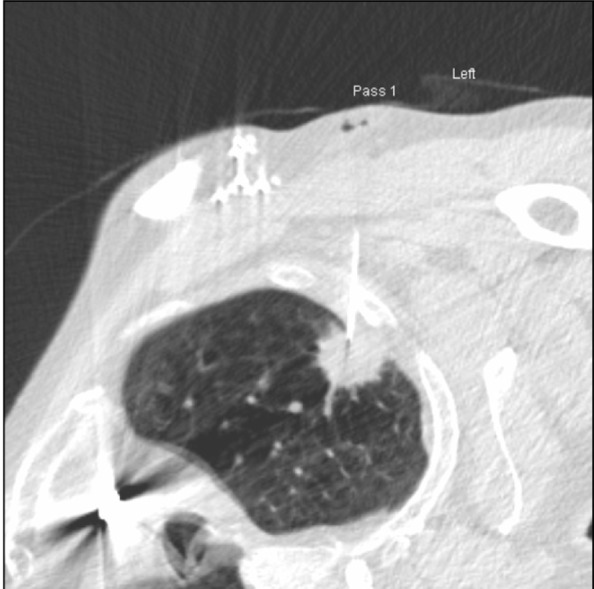
The 22g needle in situ with little or no lung involvement.

**Figure 5:**
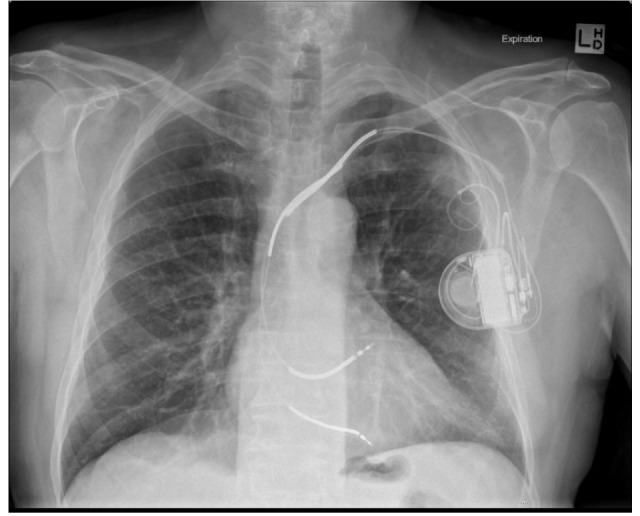
Expiratory chest radiograph taken 2 hours post-procedure shows no pneumothorax despite the patient’s history of COPD.

Using aseptic technique, the region was cleaned with Chlorhexidine and Alcohol 70% and local anaesthaetic (10 ml of Lignocaine 1%) was injected via a 23 G × 32 mm needle. An 18 G × 9 cm Angiotech “spinal” biopsy needle was inserted under CT guidance (6 contiguous images at 2.4 mm thick). A 22 G 12 cm Angiotech “spinal” biopsy needle was then introduced using a coaxial technique and guided into the lesion (Figure 4), which was aspirated using a 25 cm minimum volume extension tube and 10 ml syringe for aspiration while moving the needle in and out repeatedly. The sample was passed immediately to an on-site cytopathologist. Microscopic examination of Giemsa-type stained slides using the rapid Diff-Quik [5] technique revealed malignant cells in the sample. The patient started to express discomfort and the needle was removed. A band-aid was applied and the patient was admitted for observation. Two hours later, an expiratory PA chest radiograph was performed and no pneumothorax was seen (Figure 5).

## RESULTS AND DISCUSSION

Near the end of the procedure, the patient did express some discomfort at the biopsy site on the left and some numbness in the right arm on which he was lying. Overall, the examination was well tolerated, and provided a sample which was positive for large cell carcinoma. This was consistent with the patient’s history, previous imaging and the location of the lesion [3].

Complications arising from percutaneous FNA of lung lesions include pneumothorax (8–64%), bleeding (2–10%) and in a small number of cases, death [1]. Gohari and Haramati [1] reported in 2004 that lesion size and depth were significant factors responsible for the incidence of pneumothorax. Ko et al. [6] reported in 2001 that a shallow puncture angle through the pleura and the presence of emphysema both contributed to the incidence of pneumothorax and that emphysema in the needle path contributed to the likelihood of chest tube insertion being required.

The chance of pneumothorax in lesions of less than 2 cm in diameter is increased by 7-fold. This is thought to be due to the difficulty of placing the needle tip in a small lesion. Lesions deeper than 2 cm from the pleura have a 4-fold increased chance of pneumothorax [1]. Deeper needle penetration increases the degree of injury to the lung during the procedure. Emphysema increases the risk of leakage from punctured air cysts. It is thought that a small entry angle through the pleura creates a tear or laceration rather than the “pinhole” which is produced by passing the needle through the pleura at an angle approaching 90 degrees. This elongated entry point is more likely to result in pneumothorax formation.

While core biopsy has a far lower false negative rate than FNA (20–30%), almost all episodes of post-procedure severe bleeding are associated with core biopsy and currently FNA remains the procedure of choice [7].

The patient in this case had a 2–3 cm lesion abutting the pleura. He had a well-documented history of COPD but most significantly, the lesion lay medial to the blade of the scapula in the standard supine or prone CT scan position of arms extended above the head. Access to the lesion required unconventional positioning or a very long needle path through the lung at an acute pleural puncture angle. The right lateral decubitus position that was adopted meant using a fairly long needle path of 9.7 cm through soft tissue but more importantly traversed the pleura at approximately 70 degrees and passed directly into the lesion.

Having a cytopathologist come to the medical imaging department can be a challenge, especially in a private medical environment. Austin and Cohen [8] described a study in 1993 which clearly showed the value of on-site inspection of pathology samples. Across 55 cases they found all FNAs performed with a cytopathologist present provided adequate sample material to generate a positive diagnosis. Of the FNAs performed without a cytopathologist present only 80% provided adequate samples. Gross visual inspection of the aspirate was not as accurate for determining whether adequate material had been acquired. They also found up to twice as many passes were made during FNAs where a cytopathologist was present. In the case discussed here, the cytopathologist’s report indicated a large cell carcinoma was most likely. In this case, the detection of abnormal cells by a cytopathologist in the first sample acquired reduced both the patient’s discomfort and the risk of complications arising from further needle passes.

## CONCLUSION

Despite a number of significant risk factors, a creative approach to patient positioning resulted in a technically challenging CT-guided FNA being performed with no significant complications. An understanding of the pitfalls of this interventional procedure and thoughtful planning before its commencement strongly influenced the outcome for both diagnosis and patient aftercare.
